# Alltägliche Grenzüberschreitungen: zur Skandalisierung der klinischen Arzneimittelprüfungen des Psychiaters Roland Kuhn

**DOI:** 10.1007/s00115-022-01296-0

**Published:** 2022-05-06

**Authors:** Hanfried Helmchen

**Affiliations:** Klinik für Psychiatrie und Psychotherapie, CBF, Charité – Universitätsmedizin Berlin, Hindenburgdamm 30, 12200 Berlin, Deutschland

**Keywords:** Arzneimitteltests, Einwilligung nach Aufklärung, Antidepressive Wirkung, Fehlverhalten, Historischer Bewertungsbias, Clinical drug tests, Informed consent, Antidepressive effect, Misbehaviour, Historical bias of evaluation

## Abstract

„Testfall Münsterlingen. Klinische Versuche in der Psychiatrie, 1940–1980“ heißt der Bericht einer Untersuchungskommission, die die Regierung des Schweizer Kantons Thurgau 2016 eingesetzt hatte, nachdem mehrere Presseartikel ab 2012 die Arzneimittelprüfungen von Roland Kuhn, dem ehemaligen ärztlichen Direktor der kantonalen Anstalt in Münsterlingen, skandalisiert hatten. Der Bericht problematisiert „aus heutiger Sicht“ „feine Diskrepanzen, in alltäglichen Grenzüberschreitungen“. Solche Grenzüberschreitungen wurden vor allem in fehlender, unzureichender oder nicht belegter Aufklärung und Einwilligung der Patienten gesehen sowie bei der Anwendung der Prüfsubstanzen, die zwischen (meist) akzeptierter oder nicht abgelehnter Einnahme und getarnter oder (selten) angedrohter Applikation per Injektion variierte. Damit zeigt der Bericht einerseits die erhebliche Entwicklung des normativen Kontextes der Behandlung psychisch Kranker in den letzten 70 Jahren und vermag andererseits durch seine detaillierten Schilderungen, heutige Therapeuten für den aktuellen Kontext zu sensibilisieren. Es ist aber vor allem die Geschichte des verantwortlichen Psychiaters Roland Kuhn, des arzneimittelprüfenden Entdeckers der antidepressiven Wirkung des Imipramin. Diese Entdeckungsgeschichte wird aus sehr unterschiedlichen Perspektiven beurteilt und damit auch relativiert und ist zwischen der Beobachtung eines „Provinzpsychiaters“ und ihrer Nobelpreiswürdigkeit angesiedelt; dabei scheinen kritisch bewertete Züge der Eigenart von Kuhns Persönlichkeit auch die gelegentlich negative Tonlage des Kommissionsberichtes beeinflusst zu haben. Festzuhalten ist, dass Kuhn mit seiner qualitativen psychopathologischen Einzelfallbeobachtung die antidepressive Wirkung einer Prüfsubstanz entdeckt hat, die als Hypothese durch nachfolgende quantitative statistische Methoden verifiziert wurde.

## Abstract (en)

“Experimental case Münsterlingen: clinical trials in psychiatry, 1940–1980” is the name of the report of a control commission established by the government of the Swiss Canton Thurgau in 2016, after several articles in the press after 2012 had criticized the drug tests carried out by Roland Kuhn, the former clinical director of the cantonal mental hospital in Münsterlingen. The report discusses “fine discrepancies in everyday borderline transgressions” “from today’s viewpoint”. These borderline transgressions were seen especially in the missing, inadequate or undocumented informed consent of patients and in the usage of test substances, which varied between the (mostly) accepted or not refused intake and the camouflaged or (seldom) threatened application via injection. Thus, the report shows on the one hand, the considerable development of the normative context of treatment of mentally ill patients in the past 70 years and on the other hand, with its detailed descriptions, it can sensitize today’s therapists to the pertinent context. But most of all this is the story of Roland Kuhn, the responsible psychiatrist and the drug testing discoverer of the antidepressive effect of imipramine. This story of the discovery is judged from very differing perspectives and is thus relativized, all the way from observations of a “provincial psychiatrist” to consideration for the Nobel Prize. At the same time critically evaluated traits of Kuhn’s personality seem to have influenced the occasionally negative comments of the commission report. We should recognize, however, that with his qualitative and psychopathological individual case observations, Kuhn discovered the antidepressive effect of a test substance that as a hypothesis was verified by subsequent quantitative and statistical methods.

## Hintergrund

„Testfall Münsterlingen. Klinische Versuche in der Psychiatrie, 1940–1980“ heißt der Bericht einer Untersuchungskommission, die die Regierung des Schweizer Kantons Thurgau 2016 eingesetzt hatte, nachdem mehrere Presseartikel ab 2012^1^ die Arzneimittelprüfungen von Roland Kuhn, dem ärztlichen Direktor der kantonalen Anstalt in Münsterlingen, skandalisiert hatten [2, 3]. Der Bericht problematisiert „aus heutiger Sicht“ „feine Diskrepanzen, in alltäglichen Grenzüberschreitungen“ bei Arzneimittelprüfungen, die dort in großem Umfang durchgeführt wurden [4, S. 270]. Solche Grenzüberschreitungen wurden gesehen vor allem in fehlender, unzureichender oder nicht belegter Aufklärung der Patienten (wie auch von Mitarbeitern und Behörden) sowie bei der Applikation der Prüfsubstanzen, die zwischen (meist) akzeptierter oder nicht abgelehnter Einnahme und getarnter oder (selten) angedrohter Applikation per Injektion variierte.

Wenn der Kommissionsbericht auch viele Hinweise auf die Entwicklung des regelnden (normativen) Kontextes enthält, so lässt er in seiner auftragsgemäßen Fokussierung auf Roland Kuhn und die Münsterlinger Klinik offen bzw. es wird nur als Forschungsdesiderat erwähnt, wie verbreitet die aus heutiger Sicht kritisierte Praxis der klinischen Arzneimittelprüfung damals war – sowie ob und wie klinische Forscher ihr eigenes Vorgehen im Hinblick auf den normativen Kontext ihrer Zeit reflektierten. Da der Kommissionsbericht die im Klappentext versprochene „Verortung in der zeitgenössischen Prüfungslandschaft“ jedoch nicht enthält [5], seien vom Autor als an frühen Arzneimittelprüfungen beteiligtem Zeitzeugen weitere Hinweise zur damaligen Praxis klinischer Arzneimittelprüfungen gegeben.

## Entwicklung der therapeutischen Praxis mit psychotropen Arzneimitteln

Gegen das Elend psychisch schwerkranker Patienten – insbesondere solcher mit chronischer Symptomatik, aber auch jener mit akuten psychotischen Erregungen oder subjektiv quälenden Halluzinationen und Wahn – in den psychiatrischen Kliniken der 1950er- und auch noch 1960er-Jahre hatten Psychiater nur unzureichende und risikoreiche Behandlungsverfahren zur Verfügung [6]. In dieser Situation weckte die Einführung des Chlorpromazin (Largactil, Megaphen; [7]) als erstes Arzneimittel mit spezifisch antipsychotischer Wirkung große Hoffnungen und führte zu einer verbreiteten Aufbruchsstimmung. Erwartungsvoll gaben Psychiater ihren Patienten von der Industrie bereitgestellte Substanzen mit psychotropen Eigenschaften in therapeutischer Absicht und begannen nach kasuistisch beobachteter positiver Wirkung, potenzielle Arzneimittel auch zunehmend zu prüfen.^2^ Es öffnete sich ein neues Gebiet klinischer Forschung, die Psychopharmakotherapie. Die pharmazeutische Industrie stellte immer neue Prüfsubstanzen mit spezifisch akzentuierten psychotropen Wirkungen her^3^ und entwickelte zusammen mit Klinikern Standards für zunehmend differenziertere und kontrollierte Prüfverfahren, z. B. seit den 1960er-Jahren in den deutschsprachigen Ländern mit der Arbeitsgemeinschaft für Methodik und Dokumentation in der Psychiatrie [8]. Allmählich ergaben sich Indikationen, d. h. Krankheitsbilder, bei denen bestimmte Substanzen besonders gut wirkten. Zudem wurden im Laufe der Jahre Nebenwirkungen und auch Komplikationen beschrieben; manche traten erst nach jahrelanger Behandlung und schleichend auf, sodass sie nur verzögert als unerwünschte Arzneimittelwirkung erkannt wurden. Nach deren Kenntnis mussten die Psychiater Nutzen-Risiko-Bewertungen vornehmen, wie sie auch von Kuhn durchgeführt wurden [4, S. 151 f]. In den 1980er-Jahren begannen Kliniker eine spezielle „Arzneimittelüberwachung in der Psychiatrie“ (AMÜP) mithilfe einer Anschubfinanzierung des damaligen Bundesgesundheitsamtes (BGA) in den Psychiatrischen Universitätskliniken München und Berlin (FUB). Damals schufen Renate Grohmann und Eckhart Rüther Pharmakovigilanz-Instrumente, um schwere unerwünschte Arzneimittelwirkungen zu erfassen, zu dokumentieren und zu bewerten [9]. In den 1990er-Jahren entstand daraus in Regensburg die Arbeitsgemeinschaft Arzneimitteltherapie bei psychiatrischen Erkrankungen AGATE [10] und in Hannover das Institut für Arzneimittelsicherheit in der Psychiatrie AMSP [11]. Staatliche Kontrollen „fingen überhaupt erst gegen Ende der 1970er-Jahre an“ [12]; eine systematische Kontrolle der klinischen Prüfung und der Arzneimittelanwendung (Pharmakovigilanz) kam in Deutschland erst nach der 1986 verabschiedeten 2. Novelle zum Arzneimittelgesetz (AMG) von 1976 in Gang.^4^

## Entwicklung des normativen Rahmens der psychiatrischen Pharmakotherapie

Der normative Rahmen dieser Entwicklung beruhte zum einen auf dem uralten hippokratischen *Nichtschadensgebot*: Seine allgemeine Formulierung (§ 2 „hüten werde ich mich davor, sie (ärztliche Verordnungen) zum Schaden und in unrechter Weise anzuwenden“ [16]) konkretisierte der französische Physiologe Claude Bernard schon 1865 forschungsspezifisch in seiner Einführung zum Studium der experimentellen Medizin, indem er feststellte, dass „it is the duty and the right of the physician to perform an experiment on man whenever it can save his life, cure him, or gain him some personal benefits“. Aber Bernard bestand auch darauf „never performing on man an experiment which might be harmful to him to any extent, even though the result might be highly advantageous to science“ ([17], zit. [13]). 1948 wurde der hippokratische Eid vom Weltärztebund (WMA) mit dem Genfer Gelöbnis modernisiert, aber das Nichtschadensgebot leider nur implizit formuliert („Erhaltung und Wiederherstellung der Gesundheit meiner Patienten soll oberstes Gebot meines Handelns sein“; [18]). Diese Forderungen zum Schutz von Forschungsprobanden wurden in Deutschland durch die Entwicklung des Arzneimittelrechts zur Pflicht: Das deutsche Arzneimittelgesetz (AMG) entstand aufgrund der Römischen Verträge in einer ersten Fassung 1961. Nicht zuletzt nach der Katastrophe durch das 1957 eingeführte Thalidomid, dem ab 1961 schwere Missbildungen (Phokomelien) zugerechnet werden mussten, wurde das AMG 1964 zwecks Verbesserung der Arzneimittelsicherheit um die Pflicht zur klinischen Prüfung erweitert, bis 1971 insgesamt 17-mal geändert, 1976 in einer grundlegenden Neufassung vom Deutschen Bundestag beschlossen [19] und seitdem weitere 17-mal novelliert. Es hat die klinische Forschung erheblich geprägt.

Zum anderen wurde der normative Kontext durch die *Einwilligung* als legitimierende Voraussetzung ärztlicher Interventionen grundlegend erweitert; sie wurde seit Anfang des letzten Jahrhunderts in staatlichen Anweisungen [20] oder in Gerichtsurteilen [21] gefordert, aber erst nach 1948 allmählich relevant, da die Normen, die für die Beurteilung der in Nürnberg angeklagten nationalsozialistischen Medizinverbrechen erarbeitet und später als Nürnberger Kodex bekannt wurden, als zentrales Kriterium die freiwillige Zustimmung potenzieller Probanden enthielten [22]; und nicht zuletzt wurde ebenfalls 1948 mit der UN-Erklärung der Allgemeinen Menschenrechte insbesondere das hier Relevante der Selbstbestimmung öffentlich gemacht [23]. Später kam die durch Gerichtsurteile geforderte ausreichende Aufklärung als eine Voraussetzung der Einwilligung hinzu. Aber noch 2002 wurde im Bundestag gefragt: „Muss die Information nur gegeben werden oder muss die Informationsgeberin bzw. der Informationsgeber dafür Sorge tragen, dass sie auch verstanden wird?“ [24, S. 192]. Die Einwilligung nach Aufklärung wurde im letzten Drittel des 20. Jahrhunderts zunehmend präzisiert und zur gesetzlichen Verpflichtung entwickelt: Dieses juristische Konzept wurde in den USA seit 1972 als „informed consent“ öffentlich bekannt und ständig differenziert [25, 26],^5^ so in den Regeln zur guten klinischen Praxis (GCP). In Deutschland finden sich erstmals Ausführungen zur Einwilligung im deutschen Arzneimittelgesetz (AMG) von 1976/1978 und in der GCP der Europäischen Union von 1986; letztere erhielten erst 2004 mit der 12. Novelle zum AMG Gesetzeskraft.

Nach der gesetzlichen Festlegung der ärztlichen Pflichten zur Aufklärung und Einwilligung ab den 1970er-Jahren begann sich die Einwilligung nach Aufklärung in der klinischen Forschung parallel zur wachsenden Bewusstwerdung der nationalsozialistischen Medizinverbrechen in den 1980er-Jahren durchzusetzen; seit der Jahrhundertwende erreichte sie auch die ärztliche Praxis; hier wird die Aufklärung allerdings aus verschiedenen Gründen (Mangel an Zeit oder Kenntnis) noch nicht überall mit der vorgeschriebenen Intensität durchgeführt.

Aber schon 1964 hatte der Weltärztebund (WMA) mit der Declaration of Helsinki (DoH) ein internationales Regelwerk ethischer Kriterien für klinische Forschung veröffentlicht, das weltweit anerkannt wird. Obwohl es sich bei der DoH – wie auch bei anderen Codices – nur um Empfehlungen handelt, werden diese jedoch von der Rechtsprechung berücksichtigt und in jenen Aspekten zur Verpflichtung, die in Gerichtsurteilen ihren Niederschlag finden. Die DoH wird seitdem aufgrund neuer Erfahrungen ständigen (derzeit der 8.) Revisionen unterzogen [31]. In den 1970er-Jahren wurden erste Ethikkommissionen^6^ gebildet, die die Einhaltung des normativen Rahmens der komplexer werdenden klinischen Forschung kontrollieren sollen und diese Normen dabei auch den Forschern vermitteln können.

Diese Entwicklung zeigt, dass der normative Rahmen klinischer Forschung immer weiter differenziert wird, um neu auftretende Probleme ethisch angemessen lösen zu können. Es ist ein kontinuierlicher Prozess intensiver Wechselwirkung zwischen klinischen Forschern, Patienten, Ethikkommissionen und der durch Parlament, Judikatur und Medien vertretenen Gesellschaft, der sich über Jahrzehnte erstreckt. Die Berichterstatter haben darauf hingewiesen, dass es sich um einen dynamischen Prozess handelt, in dem sich die klinische Arzneimittelprüfung ebenso wie ihr normativer Kontext kontinuierlich differenzieren. Deshalb werfen die Wechselwirkungen zwischen beiden Prozessen bisher unbeantwortete Fragen auf, insbesondere solche, die auf die zeitlichen Verhältnisse zielen: vom Allgemeinwissen über die konkrete und spezifische Kenntnis der ethischen Regeln und ihres jeweiligen Entwicklungsstandes bis hin zu ihrer praxisbestimmenden Verbindlichkeit und Verinnerlichung.

Vermutlich begann die Einholung schriftlicher Einwilligungserklärungen erst in den 1980er-Jahren. Jedenfalls konnten in Münsterlingen erst ab dieser Zeit entsprechende Belege nachgewiesen werden. Im Allgemeinen aber wird man für diese Zeit kaum noch auf entsprechende Belege in Krankengeschichten zurückgreifen können, da diese oft nach der pflichtgemäßen Aufbewahrungsfrist von 30 Jahren vernichtet wurden. Einwilligungserklärungen zu Studien müsste man allerdings noch in den Archiven jener Firmen finden, die klinische Studien durchgeführt haben – wenn sie nicht auch dort schon vernichtet wurden. Eine weitere Quelle könnten Publikationen von Studien sein, wenn sich dort – wie jetzt üblich – Informationen über das Ergebnis der Prüfung durch die Ethikkommission finden, da davon auszugehen ist, dass Ethikkommissionen die „Informed-consent“-Unterlagen prüfen. Alles in allem bleibt der Zeitpunkt bisher im Zwielicht, ab wann die Einholung von Einwilligungserklärungen und in welcher Form (mündlich, schriftlich) begann, in der klinischen Forschung wohl eher als in der klinischen Praxis, und ab wann sie in breiter Front realisiert wurde. Denn wenn man die Standards von damals mit den heutigen vergleichen will, wäre zu wissen wichtig, ab wann klinische Forscher die forschungsrelevanten ethischen Regeln kennen mussten und ab wann sie zu ihrer Befolgung verpflichtet waren. Ohne solche Kenntnis kann der Gewinn eines epochalen Vergleichs empirischer Daten allenfalls darin liegen, den Wandel von damals bis heute als solchen zu beschreiben und dabei einen selektionsbedingten Beschreibungsbias zu reflektieren. Wenn man aber heute damaliges Verhalten ethisch bewerten will, dann muss man es zumindest auch nach den damaligen Standards beurteilen, um einen Bewertungsbias zu vermeiden.^7^

## Offene Fragen

Die zeitliche Entwicklung des normativen Kontextes von Arzneimittelprüfungen konnte nur grob skizziert werden und lässt viele Fragen offen, insbesondere zur Einwilligungsnorm; sie beziehen sich auf die Zeitpunkte der Entstehung dieser Norm, ihrer verbindlichen Gültigkeit und Umsetzung in der Praxis sowie auf ihre inhaltlichen Ausformungen: So sind Belege bereits aus den 1930er-Jahren bekannt, die als Einwilligungs- bzw. Zustimmungserklärungen bezeichnet wurden, aber mit dem heutigen „informed consent“ überhaupt nichts zu tun haben. Denn dabei handelt es sich um reine Haftungsausschlusserklärungen zum Schutz der Ärzte bzw. Krankenhäuser bei risikobelasteten Behandlungen wie der Malariatherapie oder der Elektrokrampftherapie (EKT), die zudem meist nur von den Angehörigen „geschäftsunfähiger“ Patienten unterzeichnet wurden. Beispielsweise hat Walter v. Baeyer 1951 in seinem Buch „Die moderne psychiatrische Schockbehandlung“ der Aufklärung ganze 9 Zeilen gewidmet;^8^ und ein kanadisches Consent-Formular aus dem Jahre 1956, das nichts anderes als eine Freistellung der Klinik und aller Mitarbeiter von jeglicher Haftung für potenzielle Schädigungen war [35, S. 32], verweist darauf, dass diese Auffassung offenbar weltweit verbreitet war. Von dort reicht die Entwicklungsspanne der Einwilligung nach Aufklärung und ihrer Dokumentation bis zum Ziel heutiger Aufklärung, dem Patienten bei seiner selbstbestimmten Entscheidungsfindung zu assistieren [36, 37].

So erscheint es nicht so ungewöhnlich, dass in Münsterlingen „Belege dafür (fehlten), dass man Patientinnen vor den 1980er-Jahren umfassend über die Prüfsubstanzen aufgeklärt, ihr Einverständnis eingeholt und schriftlich dokumentiert hätte. Die ersten überlieferten Einwilligungserklärungen …. stammen von 1987.“ [4, S. 112]. Roland Kuhn müsste zwar die für die Schweiz seit 1970 empfohlenen Regeln der für die Teilnahme an klinischen Forschungsuntersuchungen empfohlenen Einwilligung nach angemessener Aufklärung der Probanden in den 1970er-Jahren [15] gekannt bzw. als Prüfer spätestens (wann?) von der pharmazeutischen Industrie zur Kenntnis erhalten haben; aber möglicherweise hat er sie als bürokratischen Formalismus angesehen und nicht beachtet, weil er von seiner eigenen ärztlich-moralischen Haltung überzeugt war – wie er sie z. B. in Nutzen-Risiko-Abschätzungen in einzelnen Krankengeschichten detailliert dokumentiert hat. Diese Möglichkeit führt zu der Frage, wie verbindlich diese Richtlinien der Schweizerischen Akademie der Medizinischen Wissenschaften (SAMW) waren bzw. ob ihre Nichtbeachtung sanktioniert wurde?

## Zur Kritik der Münsterlinger Arzneimittelversuche

Vor diesem fragwürdigen Hintergrund stellen sich Fragen zur ethischen Beurteilung der Münsterlinger Versuche und des für sie verantwortlichen klinischen Forschers Roland Kuhn. Zunächst ist davon auszugehen, dass der Kommissionsbericht eine Prüfpraxis in der Münsterlinger Klinik beschreibt, die sich in den 1950er- und 1960er-Jahren wohl kaum von der Prüfpraxis in anderen Kliniken unterschied. Denn zum einen ist in den 1950er-Jahren keine Kritik am methodischen Vorgehen Kuhns bekannt geworden und zum anderen führte erst im Laufe der folgenden Jahrzehnte die kritische Auseinandersetzung mit ethischen wie auch prüfungstechnischen Problemen dazu, dass jene Grenzen definiert und präzisiert wurden, die den normativen Rahmen heutiger Arzneimittelprüfungen bestimmen.^9^ Dem Kommissionsbericht ist nicht nur zu entnehmen, wie sich das Umfeld klinischer Forschung grundlegend gewandelt hat und dazu mehrerer Generationen bedurfte, sondern er sensibilisiert auch heutige Forscher dafür, diese sehr detailliert aufgezeigten und heute geltende normative Grenzen wahrzunehmen und einzuhalten. Obwohl der Bericht insgesamt eher vorsichtig (z. B. im Konjunktiv) formuliert, erscheint jedoch ein gelegentlich vorwurfsvoller Ton gegenüber Roland Kuhn nicht berechtigt.

Auch blieb Kuhn bei seiner von der Zeit überholten Prüfmethode nicht einfach stehen; vielmehr folgte er nicht dem ab den 1960er-Jahren aufkommenden Mainstream quantitativer Prüfverfahren [40], sondern behielt seine qualitative Prüfpraxis der psychopathologischen Einzelfallbeobachtung bei; jedoch nicht aus Unvermögen, sondern weil er von seiner Methode überzeugt war und sie vehement verteidigte [4, S. 152 f]. Denn nicht zuletzt damit hatte er die antidepressive Wirkung des Imipramin entdeckt und damit einen für einen „Provinzpsychiater“ ungewöhnlichen Erfolg internationaler Anerkennung erzielt – bis hin zu Anmerkungen über ihre Nobelpreiswürdigkeit [41]. Diese Entdeckung und ihre Verteidigung lassen Eigenheiten seiner Persönlichkeit hervortreten, die letztlich als Hintergrund kritischer Vermutungen und herabsetzender Formulierungen erscheinen. Damit ist der Kommissionsbericht auch die Geschichte des Arzneimittelprüfers Kuhn bzw. des Einflusses der kritisch bewerteten Züge seiner Persönlichkeit auf die Tonlage des Berichts.

## Roland Kuhn als Entdecker

Roland Kuhn (1912–2005) wollte Chirurg werden, absolvierte jedoch eine psychiatrische Weiterbildung bei Jacob Klaesi,^10^ war als Experte für den Rorschach-Test bekannt geworden [42], erlernte die Daseinsanalyse bei deren Schöpfer Ludwig Binswanger d. J. und hatte weitgespannte geisteswissenschaftliche, insbesondere philosophische Interessen. Die Phänomenologie Husserls schärfte seinen psychopathologischen Blick. Ende der 1940er-Jahre beschäftigte er sich nach Hinweisen von Binswanger, aber auch mit der Möglichkeit medikamentöser Behandlung der Depression; dies war für ihn naheliegend, da die antidepressive Wirkung der lange bekannten Opiumkur und der Elektrokrampftherapie offensichtlich durch Einwirkung auf das Gehirn, also biologisch determiniert erschien – so wie dies auch von einer antidepressiven Medikation zu erwarten war [43, 44].

Im Jahr 1957 beschrieb er – der bereits seit 1946 klinische Prüfungen potenzieller Arzneimittel durchgeführt hatte – die antidepressive Wirkung einer ihm von der pharmazeutischen Firma GEIGY zur klinischen Prüfung angebotenen chemischen Prüfsubstanz. Der Weg zur Entdeckung dieser antidepressiven Wirkung wird jedoch durch sehr unterschiedliche Erinnerungen beschrieben, so insbesondere zum einen von GEIGY-Mitarbeitern als Zufallsentdeckung, zum anderen von Kuhn als Ergebnis systematischer klinischer Einzelfallbeobachtung. Mit dieser Entdeckung der spezifisch antidepressiven Wirkung einer trizyklischen psychotropen Substanz war er ein Begründer der antidepressiven Arzneimitteltherapie.^11^

Nachdem 1952 die antipsychotische Wirkung des Chlorpromazin (Largactil, Megaphen) bekannt wurde [47, 48], wandten sich Mitarbeiter von GEIGY an Kuhn, den sie bei einer Prüfung bereits 1950 als „an extremely perceptive clinical observer“ [40, S. 72] kennengelernt hatten. Damals hatte Kuhn ein Antihistaminikum auf eine schlaffördernde Wirkung geprüft. Die hypnotische Wirkung war jedoch zu gering, sodass die Prüfung abgebrochen wurde. 1952 bat GEIGY Roland Kuhn nun, ein weiteres Antihistaminikum, das trizyklische Iminodibenzyl (Prüfnummer G 22355), ein Analogon des Chlorpromazin (gleiche Seitenkette am trizyklischen Phenothiazinring), auf eine antipsychotische Wirkung bei schizophren Kranken zu prüfen.^12^ Da es zu dieser Zeit noch keine Kontrollmethoden der klinischen Prüfung gab, musste ein Effekt sehr deutlich in Erscheinung treten, um als bedeutsam anerkannt zu werden. Dies war tatsächlich der Fall. Nur unterschied sich die Wirkung sehr eindrücklich von der antipsychotischen Wirkung des Chlorpromazin. Denn nach einigen Tagen wurden etliche Patienten, insbesondere jene mit einer depressiven Begleitsymptomatik unruhig oder hypomanisch. Dieses zunächst enttäuschende Ergebnis führte zu vielen Diskussionen zwischen GEIGY-Mitarbeitern, insbesondere Paul Schmidlin und Roland Kuhn. Dabei sei die Idee entstanden, G22355 bei depressiven Patienten zu prüfen. 1955 begann Kuhn mit dieser Prüfung; schon die ersten drei Patienten zeigten eine dramatisch antidepressive Wirkung, bei zwei Dritteln der dann vierzig geprüften Patienten wurde die depressive Symptomatik deutlich reduziert [40]. Die antidepressive Wirksamkeit des nun Imipramin (generischer Name) bzw. Tofranil (Handelsname) genannten Arzneimittels wurde durch andere Untersucher bestätigt, ab 1960 in zunehmend kontrolliert durchgeführten Prüfungen. Bis 1970 erschienen mehr als 4000 Publikationen zu Imipramin [40].

Diese Sicht der GEIGY-Mitarbeiter kränkte Roland Kuhn sehr, denn er sah sich allein als Entdecker der antidepressiven Wirkung des Imipramin [50, 39]. Diese Entdeckung sei auch kein Zufall („serendipity“), sondern Ergebnis intensiver klinisch-psychopathologischer Beobachtung des individuellen Patienten gewesen. Nur so habe er als zentrale Indikation des Imipramin das „vegetative Syndrom“ (Appetit‑, Schlaf‑, Sexualstörungen, psychomotorische Hemmung, Denkhemmung und Entscheidungsschwäche sowie Tagesschwankungen) der (endogenen) Depression erkennen können [51]. Diese Erkenntnis Kuhns wird auch nicht dadurch entwertet, dass er dazu von einem Assistenzarzt schriftlich fixierte Beobachtungen des Pflegepersonals beizog [4] S. 88; [52]. Sowohl in der ersten Publikation über Imipramin (Kuhn 1957 [53]) als auch nach zitierten Unterlagen im Kommissionsbericht beschrieb Kuhn die klinischen Wirkungen der Versuchspräparate präzise und differenziert und bewertete sie kenntnisreich. Kuhn erscheint als fleißiger, sehr präsenter Psychiater, der klinisch genau beobachtete und fast zwanghaft dokumentierte. Er zeigte sich aber auch als äußerst selbstgewisse Persönlichkeit; immer überzeugt, das Richtige getan zu haben, reagierte er auf Kritik gelegentlich ebenso empfindlich wie heftig. Auch schien er sehr darauf bedacht, die Entdeckung der antidepressiven Wirkung von Imipramin bei „vitalen“ (endogenen) Depressionen allein auf seine klinisch-psychopathologische Kompetenz zurückzuführen und die Mitwirkung weiterer Personen, so des Freiburger Klinikers Clemens Faust und des Basler Psychopharmakologen Paul Schmidlin von GEIGY, unerwähnt zu lassen [40, 54]. Asketisch streng führte er die Klinik hierarchisch-paternalistisch; sehr häufig betonte er etwas als „absolute“, „not at all“; weder Zweifel noch auch nur ein Zögern waren zu bemerken. Ich habe Roland Kuhn wohl zwei- oder dreimal bei Tagungen als eine Persönlichkeit erlebt, die mich in ihrer selbstgewissen und missionarisch eifernden Art eher unangenehm beeindruckte. Diese autoritäre Haltung kam nicht bei allen Mitarbeitern gut an und führte zu gelegentlichen Meldungen über als unzulässig wahrgenommene Aspekte der Prüfungen an die Öffentlichkeit oder zuständige Behörden – und sie scheint auch die Berichterstatter zu einem gelegentlich herabsetzenden Ton verführt zu haben. Auch mag Kuhns klinische Methodik manchem späteren Beobachter chaotisch erscheinen, brachte aber mit der Entdeckung der antidepressiven Wirkung eines Arzneimittels einen Durchbruch, der die Behandlung der Depression nachhaltig beeinflusste und für unsere Strategien zur Arzneimittelentwicklung nutzbringend berücksichtigt werden könnte [55]. Seine als unsystematisch „impressionistisch“ [4, S. 153] abgewertete qualitative Prüfmethode der psychopathologischen Einzelfallbeobachtung verteidigte Kuhn mit schärfster Kritik an den ab 1960 zunehmend kontrollierten Prüfungen, indem er mehrfach äußerte „I have never used ‚controlled double blind studies‘ with ‚placebo‘, ‚standadized rating scales‘ or ‚statistical treatment of data based on a large number of patients‘“ [51, S. 76]. Der kanadische Psychiatriehistoriker Edward Shorter kommentierte diesen Dissenz 2017: „We owe Roland Kuhn … the validation of the ‚clinical method‘, as opposed to the ‚statistical method‘, in drug discovery… Yet the numbers turned out to be so vulnerable to commercial spin and manipulation that it is not entirely clear, even today, whether the current crop of ‚antidepressants‘, the SSRIs, are actually effective in real depression, though they may be effective in nervous illnesses of various kinds“ [56].

## Footnotes

1 Das Jahr seines 100. Geburtstages [1]

2 Diese therapeutische Intention bei individueller Anwendung eines (noch) nicht zugelassenen Arzneimittels wird als *Heilversuch* bezeichnet. Er wurde im Lauf der Jahre aus therapeutischen Gründen differenziert bzw. erweitert um den Off-Label-Use und den Compassionate-Use (https://de.wikipedia.org/wiki/Heilversuch). In der klinischen Praxis ist die Grenze zwischen therapeutischer und erkenntnissuchender Intention nicht immer klar; diese Unterscheidung hat aber heute an Bedeutung verloren, da der Patient in jedem Falle angemessen aufgeklärt werden muss.

3 Gelegentlich in Zusammenarbeit mit Klinikern, die dann auch um Zuweisung von Prüfsubstanzen baten: „Antragsforschung“ gegenüber der später üblichen reinen „Auftragsforschung“, in der Kliniker ausschließlich von der Industrie entwickelte Substanzen nach nur noch von ihr aufgestellten Prüfplänen prüften.

4 In den USA wurde schon 1962 mit dem Kefauver-Harris-Act festgelegt, dass Arzneimittel zum Markt nur zugelassen werden, wenn sie sich zuvor in klinischen Prüfungen als wirksam und sicher erwiesen haben [13]; 1966 fügte die US-amerikanische Zulassungsbehörde FDA (Food and Drug Administration) ihren Regeln die Verpflichtung hinzu, dass vor jeder Arzneimittelprüfung die Probanden nach Aufklärung einwilligen müssen [14]. Die Schweizer Akademie der Medizinischen Wissenschaften (SAMW) publizierte erstmals 1970 Regeln zur ethischen Bewertung von „Forschungsuntersuchungen am Menschen“ [15], deren Fortschreibungen 1981, 1989, 1997 Grundlage für das 2011 beschlossene Schweizer Gesetz zur Forschung am Menschen waren.

5 Der amerikanische Anästhesist Henry Knowles Beecher fand bei der Analyse von 100 Publikationen über klinische Forschungsuntersuchungen 22 als ethisch fragwürdig oder nicht akzeptabel [27]. Mit dieser Publikation setzte Beecher 1966 eine Diskussion zur Ethik in der medizinischen Forschung in Gang, die sich nach der großen öffentlichen Resonanz der 1972 bekannt gewordenen Tuskagee-Studie [28, 29] zu einer grundlegenden bioethischen Bewegung entwickelte und damit den Kontext ärztlichen Handelns verwandelte: im Bulletin der WHO wurde Beechers Publikation charakterisiert1 als „the most influential single paper ever written about experimentation involving human subjects“ and thereby „played a significant role“ in enacting strict federal regulations [30].

6 Die Deutsche Forschungsgemeinschaft (DFG) begann in den 1970er-Jahren ihre Mittelzuteilung für Sonderforschungsbereiche an die Existenz von Ethikkommissionen zu knüpfen [32]. Entsprechende Institutional Review Boards (IRBs) wurden in den USA bereits 1965 vorgeschrieben [13].

7 „Es ist allerdings sehr die Frage, ob das heute weit verbreitete Verfahren, Maßstäbe von heute rücksichtslos rückwirkend auf gestern anzuwenden, irgendeinen relevanten Erkenntnisgewinn bringt, oder etwa nur der moralischen Selbstvergrößerung und Überheblichkeit dient,“ [33].

8 „Um sich vor ungerechtfertigten Anzeigen zu schützen, tut man gut, sich von den nächsten für den meist geschäftsunfähigen Kranken verantwortlichen Angehörigen einen Revers unterschreiben zu lassen. Der Revers soll die Form einer kurzen Zustimmungserklärung haben, in der auf die praktisch geringe Gefährdung durch Gesundheitsschädigungen aufmerksam gemacht wird. Ausführliche schriftliche oder mündliche Ausführungen über alle denkbaren Komplikationen oder Zwischenfälle zu geben, ist unnötig und im Interesse der Kranken unzweckmäßig. Die Zustimmung würde dann allzuoft verweigert werden.“ [34, S. 14].

9 Diese sind inzwischen so detailliert und fordern so hohe Standards ein, dass pharmazeutische Firmen Schwierigkeiten haben, in entwickelten Ländern klinische Prüfer und den Standards entsprechende Probanden zu finden [38].

10 Lehrstuhlinhaber für Psychiatrie in Bern, der 1920 eine medikamentös induzierte Schlafkur gegen Schizophrenie einführte.

11 Ebenfalls 1957 schlug Nathan Kline den als Tuberkulostatikum angewandten Monoaminooxidasehemmer Iproniazid (Marsilid) als Antidepressivum vor, nachdem bereits 1952 im Tierversuch eine antidepressive Wirkung des Strukturanalogons Isoniazid beobachtet worden war [45, 46].

12 Sedierung ist eine unerwünschte Nebenwirkung vieler Antihistaminika [49]. Als schlafinduzierende erwünschte Hauptwirkung enttäuschte sie. Aber dem französischen Militärchirurgen Henri Laborit fiel bei seinen Versuchen mit einem lytischen Cocktail zur Narkoseeinleitung auf, dass der Zusatz des Antihistaminikums Chlorpromazin nicht nur eine leichte Sedierung, sondern auch ein „entspanntes Desinteresse“ bewirkte; deshalb regte er den Psychiater Jean Delay und Mitarbeiter an, Chlorpromazin gegen psychische Störungen zu prüfen. Sie stellten eine spezifisch antipsychotische Wirkung des Chlorpromazin fest [7]. Damit begann die Ära einer spezifisch antipsychotischen Pharmakotherapie.
